# The role of vasopressin in olfactory and visual processing

**DOI:** 10.1007/s00441-018-2867-1

**Published:** 2018-06-27

**Authors:** Douglas Wacker, Mike Ludwig

**Affiliations:** 1 School of STEM (Division of Biological Sciences), University of Washington Bothell, Bothell, WA USA; 2 Centre for Discovery Brain Sciences, University of Edinburgh, Edinburgh, UK; 3 Centre for Neuroendocrinology, University of Pretoria, Pretoria, South Africa

**Keywords:** Vasopressin, Olfaction, Retina, Social behaviour, Olfactory bulb

## Abstract

Neural vasopressin is a potent modulator of behaviour in vertebrates. It acts at both sensory processing regions and within larger regulatory networks to mediate changes in social recognition, affiliation, aggression, communication and other social behaviours. There are multiple populations of vasopressin neurons within the brain, including groups in olfactory and visual processing regions. Some of these vasopressin neurons, such as those in the main and accessory olfactory bulbs, anterior olfactory nucleus, piriform cortex and retina, were recently identified using an enhanced green fluorescent protein-vasopressin (eGFP-VP) transgenic rat. Based on the interconnectivity of vasopressin-producing and sensitive brain areas and in consideration of autocrine, paracrine and neurohormone-like actions associated with somato-dendritic release, we discuss how these different neuronal populations may interact to impact behaviour.

In addition to its well characterized role as a neurohypophyseal hormone, vasopressin (or its non-mammalian homologue, vasotocin) acts within the brain to modulate circadian rhythmicity, social recognition, aggression, affiliation and other social behaviours in vertebrates (Buijs and Swaab 1979; Kelly and Goodson 2014; Terranova et al. 2017; Tsuji et al. 2017b; Wacker and Ludwig 2012). Vasopressin can be released in multiple ways: (1) peripherally from axon terminals of magnocellular hypothalamic neurons into the systemic circulation via the posterior pituitary; (2) into the hypophysial blood portal system, bound for the anterior pituitary to potentiate adrenocorticotropic hormone (ACTH) release; (3) within the brain, from the axonal varicosities of centrally projecting neurons and (4) from the soma and dendrites of vasopressin neurons to induce extrasynaptic autocrine/paracrine effects and neurohormonal-like actions on more distant targets (Ludwig and Leng 2006; Leng and Ludwig 2008). There is no evidence that circulating vasopressin can pass the blood-brain barrier in appreciable amounts, so any behavioural effects of neurohypophyseal vasopressin are likely the indirect result of changes to peripheral physiology, like blood pressure via action on blood vessels and the kidney (Mens et al. 1983; Reppert et al. 1981). Axonal and dendritic vasopressin release can be differentially modulated; the dendrites of vasopressin neurons in the supraoptic nucleus (SON), paraventricular nucleus (PVN) and suprachiasmatic nucleus (SCN) all contain abundant vasopressin vesicles and are likely to be the main source of intrahypothalamic vasopressin release (Leng and Ludwig 2008; Ludwig and Leng 2006; Ludwig and Stern 2015). Vasopressin is always co-localised with a conventional transmitter, so vasopressin neurons also have roles that do not always involve vasopressin release. Vasopressin induces its effects in mammals by binding to three vasopressin receptors, the vasopressin 1a (V1aR), vasopressin 1b (V1bR) and vasopressin 2 (V2R) receptors (Birnbaumer 2002). V1aR is found in the brain and vascular smooth muscle, V1bR in the brain and anterior pituitary and V2R in collecting duct cells of the kidneys but not in the brain. Vasopressin can also signal by activating oxytocin receptors (Song and Albers 2017).

Behavioural regulation by vasopressin begins with the modulation of sensory processing and integration centres, which, in turn, wire into larger regulatory networks, such as the social-decision-making network, to facilitate the execution of context-appropriate behaviour (O'Connell and Hofmann 2011; Wacker and Ludwig 2012). There are multiple sources of vasopressin in the brain, including some more recently described vasopressin cells in the olfactory bulb, anterior olfactory nucleus (AON), piriform cortex (PIR) and retina of the rat (Tobin et al. 2010; Tsuji et al. 2017a, b; Wacker et al. 2010). These newly described cells were identified using an enhanced green fluorescent protein-vasopressin (eGFP-VP) transgenic rat in which the gene for GFP was spliced into exon 3 of the vasopressin preprogene, which normally codes for copeptin, which has no known biological function (Ueta et al. 2005). Driven by the vasopressin promoter, eGFP transgene expression results in the production of eGFP in vasopressin cells. It is possible that some eGFP is expressed in cells that do not normally synthesize vasopressin in eGFP-VP rats. However, immunohistochemical double-labelling has confirmed the co-localization of eGFP and vasopressin in the olfactory bulbs and AON and eGFP and vasopressin-associated neurophysin in the PIR and retina, the latter of which was confirmed by PCR showing vasopressin mRNA expression (Tobin et al. 2010; Tsuji et al. 2017a, b; Wacker et al. 2010). Also, the SON and PVN show predicted increases in eGFP mRNA expression with dehydration, while the SCN does not, as would be predicted based on the physiological responses of typical vasopressin neurons (Tsuji et al. 2016, 2017b; Ueta et al. 2005). Work examining how different neural and peripheral vasopressin signalling pools interact to modulate social behaviour will help clarify potential mechanisms of action that may be targeted in translational studies. Here, we review the effects of vasopressin on neural olfactory and visual processing regions and give examples of how vasopressin signalling across neural areas mediates changes in vertebrate social behaviour. Many, but not all, neural vasopressin signalling systems show some level of sexual dimorphism, whether in the peptide itself or its receptors (Dumais and Veenema 2016). Vasopressin studies reviewed here were conducted using male animals, except where noted otherwise.

## Vasopressin and odour processing

Vasopressin-producing neurons have been described in multiple brain regions associated with the processing of olfactory and pheromonal signals (Caffe and Van Leeuwen 1983; Shepherd et al. 2003; Tobin et al. 2010; Tsuji et al. 2017a; Wacker et al. 2010). Chemosensory information is transmitted across two processing streams, the main and accessory olfactory systems. Although these systems were once considered relatively segregated, it is now clear that there are multiple locations where crosstalk and integration can occur (Keshavarzi et al. 2015; Martinez-Marcos 2009; Yamaguchi 2017). Olfactory sensory neurons of the main olfactory epithelium (MOE) receive volatile, non-social odours and in many mammals, including mice and rats, non-volatile pheromones are received by vomeronasal sensory neurons of the vomeronasal organ (VNO), though these neurons can also transduce volatile odorant information (Leinders-Zufall et al. 2000; Shepherd et al. 2003; Trinh and Storm 2003). Recent work also suggests that pheromonal signalling can occur through the MOE with subsequent processing via the main olfactory system (Wang et al. 2006; Xu et al. 2005).

**Fig. 1:**
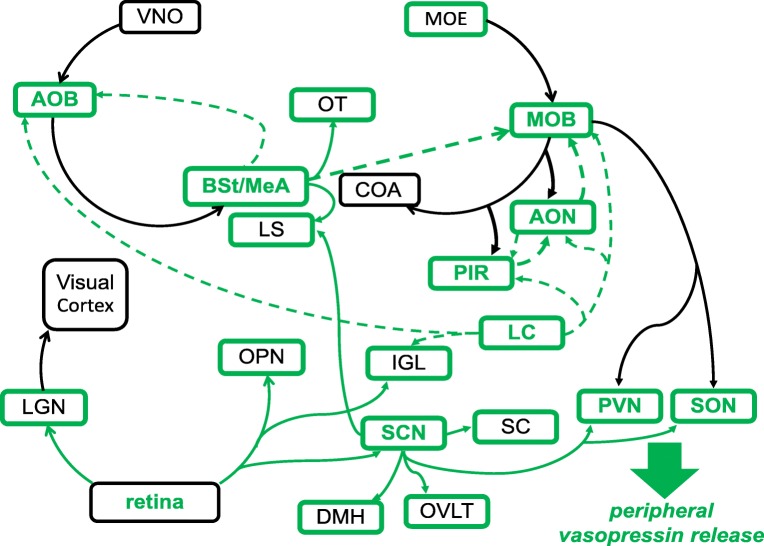
Functional wiring diagram of vasopressin connections in olfactory and visual processing regions in the rat. Brain regions are not arranged topographically, rather by functional group. The upper left represents the accessory olfactory system, the upper right represents the main olfactory system and the lower region represents visual/light processing. Regions containing vasopressin neurons have green lettering. Vasopressin-sensitive regions (V1aR or V1bR expression and/or vasopressin binding via autoradiography) have green outlines. Known vasopressin connections appear as solid green lines (e.g., retina to LGN), potential but unconfirmed vasopressin connections appear as dotted green lines and non-vasopressin connections appear as solid black lines. For clarity, not all non-vasopressin connections are displayed. Regions containing vasopressin cell bodies, dendrites and axons may also exert local paracrine and autocrine, as well as more distant neurohormone-like effects and therefore represent an important signalling mechanism not fully represented in axonal wiring diagrams. See text for pertinent connectivity citations. See Table 1 for key to brain region abbreviations

Olfactory and vomeronasal sensory neurons terminate in glomeruli of the main (MOB) and accessory olfactory bulbs (AOBs) respectively, where olfactory information undergoes complex processing and integration by multiple intrinsic and extrinsic cell types (Shepherd et al. 2003). Odour information, coded by the activation of particular glomeruli, is sent from the mitral cells and a subset of tufted cells along the lateral olfactory tract towards higher processing areas. Information from the MOB is forwarded to a variety of structures, including the AON, PIR, olfactory tubercle (OT) and anterior (COAa) and posterior lateral subdivisions of the cortical amygdala (COApl) (Scalia and Winans 1975; Scott et al. 1980). The MOB also projects to SON, home to magnocellular vasopressin neurons, in male and female rats (Meddle et al. 2000; Smithson et al. 1989) and recent work in mice detected a connection from the MOB to vasopressin neurons of both the SON and PVN (Bader et al. 2012b). Information from the AOB is routed directly to the bed nucleus of the stria terminalis (BSt), medial amygdala (MeA) and posterior medial subdivision of the cortical amygdala (COApm). Work over the last decade has also demonstrated direct projections from the MOB to the MeA, representing a connection between the main and accessory olfactory systems (Bader et al. 2012a; Kang et al. 2009, 2010). The MOB in the rat receives input from multiple brain regions, including the AON, PIR, OT, locus coeruleus (LC), nucleus of the lateral olfactory tract (nLOT), COAa, COApl and multiple hypothalamic areas (de Olmos et al. 1978; McLean et al. 1989). The AOB receives information from neurons in the BSt, MeA, COApm, LC and bed nucleus of the accessory olfactory tract (BAOT). Vasopressin and its central receptors are expressed at a surprisingly large number of locations across both olfactory processing pathways, including the MeA and BSt, which are known mediators of social behaviour. Many vasopressin-producing and vasopressin-sensitive brain regions are interconnected, potentially allowing for complex modulation of olfactory information, including social signals, by this neuropeptide (Fig. 1).

**Table 1:** Abbreviations of brain regions referenced in this review

Abbreviation	Brain region
AOB	Accessory olfactory bulb
AON	Anterior olfactory nucleus
BAOT	Bed nucleus of the accessory olfactory tract
BSt	Bed nucleus of the stria terminalis
COA	Cortical amygdala
COAa	Cortical amygdala (anterior part)
COApl	Cortical amygdala (posterior part, lateral zone)
COApm	Cortical amygdala (posterior part, medial zone)
DMH	Dorsomedial nucleus of the hypothalamus
En	Endopiriform nucleus
IC	Islands of Calleja
IGL	Intergeniculate leaflet
LC	Locus coeruleus
LGN	Lateral geniculate nucleus
LS	Lateral septum
MeA	Medial amygdala
MOB	Main olfactory bulb
MOE	Main olfactory epithelium
nLOT	Nucleus of the lateral olfactory tract
OPN	Olivary pretectal nucleus
OT	Olfactory tubercle
OVLT	Organum vasculosum laminae terminalis
PIR	Piriform cortex
PVN	Paraventricular nucleus
SC	Superior colliculus
SCN	Suprachiasmatic nucleus
SON	Supraoptic nucleus
VNO	Vomeronasal organ

### Main olfactory epithelium and vomeronasal organ

V1aR mRNA is expressed in rat globose basal cells, the primary neural stem cells of the MOE (Caggiano et al. 1994; Levasseur et al. 2004). Cultured olfactory epithelium cells show a dose-dependent increase in intracellular calcium release in response to vasopressin administration (Levasseur et al. 2004). More work needs to be completed to determine the functional relevance of this signalling, as well as the source, potentially local, of vasopressin in this region. With the recent description of steroid-binding globulins within the main olfactory epithelium, it would be informative to examine co-localization and steroid-sensitivity of V1aR in this area, considering the dependence of some other vasopressin-signalling regions on gonadal steroids (Caldwell et al. 2017; Ploss et al. 2014). Unlike in the MOE, neither vasopressin nor its receptors have been identified in the VNO. Bluthé and Dantzer (1993) reported that ablation of the VNO eliminated the ability of a subcutaneously applied V1R antagonist to block short-term social recognition in male rats. However, deficits in such recognition induced by removal of the VNO were only temporary and social recognition can be blocked by inhibition of vasopressin signalling exclusively within the MOB, suggesting that the VNO is not required (Bluthé and Dantzer 1993; Tobin et al. 2010).

### Main and accessory olfactory bulbs

Vasopressin neurons are present in the main (MOB) and accessory olfactory bulbs (AOBs) of male and female rats and V1aR and V1bR expression and/or binding has been described in the olfactory bulbs of a variety of rodents (Beery et al. 2008; Campbell et al. 2009; Corbani et al. 2017; Litvin et al. 2011; Tobin et al. 2010). Recent work has also identified vasotocin, the non-mammalian homologue of vasopressin, in the olfactory bulbs of male Mozambique tilapia fish, V1aR-immunoreactivity and mRNA (V1a2R) in the olfactory bulbs of male and female rock hind and male Burton’s mouthbrooder fish and vasotocin receptor 4 (VT4R, akin to mammalian V1aR) in the olfactory bulbs of the chicken (Almeida et al. 2012; Huffman et al. 2012; Kline et al. 2011; Selvam et al. 2013). Bulbar vasopressin cells have been best characterized in male and female rats, which based on their neurochemical identity (glutamatergic but not GABAergic), location in the external plexiform layer, electrophysiological properties (bursting) and lack of extrabulbar projections have been identified as a subset of external tufted cells that lie entirely within the olfactory bulb (Leng et al. 2014; Tobin et al. 2010). Axons of intrinsic external tufted cells terminate in the internal plexiform layer (immediately adjacent to the mitral cell layer) and granule cell layer (Shepherd et al. 2003; Tobin et al. 2010). Vasopressin-producing external tufted cells extend their primary dendrites into neighbouring glomeruli, which receive olfactory information from olfactory sensory neurons (Tobin et al. 2010).

Some bulbar vasopressin neurons co-express V1bR (but not V1aR), suggesting potential para/autoregulation (Tobin et al. 2010; Wacker et al. 2011). Vaccari et al. (1998) showed that V1bR mRNA is robustly expressed in the mitral cell layer and to a lesser extent in the external plexiform layer of the MOB of male rats and immunohistochemical studies have revealed V1aR- and V1bR-ir in the glomerular and mitral cells layers of both males and females (Tobin et al. 2010). It is therefore possible that both axonal (to mitral cell bodies) and dendritic (to mitral cell dendrites) release of vasopressin plays a role in the processing of olfactory information (Wacker et al. 2011; Wacker and Ludwig 2012). In fish, V1aR are expressed primarily in the granule cell layer (Huffman et al. 2012; Kline et al. 2011), while in the chicken, V1aR-like receptors are found on glial cells, likely tanycytes, lining the olfactory ventricle (Selvam et al. 2013).

In rats, vasopressin application reduces mitral cell firing in the MOB (Tobin et al. 2010). Blocking this inhibition, whether via induced vasopressin cell death, V1aR antagonism or V1aR gene silencing, abolishes short-term social recognition in male rats. We hypothesize that upon first exposure to and olfactory investigation of a conspecific, large dense core vesicles are mobilized in vasopressin dendrites in the periglomerular region of the olfactory bulb (Wacker and Ludwig 2012). If re-exposure to the now familiar conspecific occurs within ~ 45 min, activation of now primed neurons elicits somato-dendritic vasopressin release into glomeruli that represent the odour profile of the sensed animal. This vasopressin binds V1aR and/or V1bR on mitral cell dendrites, thereby inhibiting an output signal to higher processing areas. Whereas initial exposure to the conspecific induces heightened olfactory investigation, such behaviour is blocked upon re-exposure by vasopressin and the focal male does not waste valuable energy reinvestigating a familiar social stimulus. This is all done without longer-term memory storage required for true individual recognition. Recent in vitro work in the mouse suggests a different and possibly complimentary mechanism for modulation by vasopressin in the AOB (Namba et al. 2016). Activated mitral cell dendrites release glutamate that activates granule cell dendrites, which then feedback on those mitral cells with GABA and inhibit their output. In a slice preparation, V1aR activation induces a dose-dependent long-term potentiation in granule cell dendrites and reduces IPSCs in mitral cells. This suggests that vasopressin may facilitate mitral cell signalling at the level of the AOB. The AOB also sends and receives projections to/from kisspeptin-immunoreactive (−ir) neurons of the posterior dorsal MeA, of which around 10% receive amygdalar vasopressin input, suggesting that vasopressin may exert indirect effects on AOB function (Pineda et al. 2017). Vasopressin produced in the olfactory bulbs alters odour processing and thereby short-term social recognition in rodents and vasopressin released from other regions, such as the AON, PIR and LC may also contribute to this processing both within and outside the olfactory bulbs.

### Anterior olfactory nucleus

The anterior olfactory nucleus (AON) is a cortical region that integrates olfactory information sent from the MOB with input from the PIR, multiple regions of the amygdala and other brain regions (Brunjes et al. 2005; Lei et al. 2006). It is divided into the pars externa and pars principalis, which is further subdivided into multiple subdivisions (Brunjes et al. 2005). The AON is interconnected with the MOB and PIR, projects to the OT (including the islands of Calleja) and receives inputs from multiple areas, including the BSt and nLOT. Vasopressin neurons, as well as cells immunoreactive for V1aR and V1bR, are found in the pars externa and all subdivisions of the pars principalis in male and female rats (Wacker et al. 2010). Vasopressin neurons in the rat AON typically co-express V1aR and always co-express V1bR, so like in the olfactory bulb, autocrine/paracrine signalling by vasopressin is likely important in processing olfactory information in the AON (Wacker et al. 2010).

Exposure to a conspecific juvenile increases neuronal activation, as assessed by the immediate early gene Egr-1, in the lateral subdivision of the pars principalis and trimethylthiazoline (TMT), a component of fox urine, increases Egr-1 in both lateral and dorsal subdivisions of the AON of male and female rats, suggesting a role for this cortical region in the processing of both conspecific and heterospecific signals (Wacker et al. 2010). Only exposure to a conspecific juvenile increases the ratio of activated to total vasopressin neurons in the lateral and dorsal AON, so vasopressin signalling in this region appears to be preferentially involved in the processing of conspecific cues. Electrical stimulation of the lateral olfactory tract increases immediate early gene expression in the dorsal AON but does not increase the number of activated vasopressin cells in any AON subdivision (Tsuji et al. 2017a). Vasopressin cells in the AON are also GABAergic, so likely represent interneurons rather than principal (output) neurons (Kay and Brunjes 2014; Wacker et al. 2010). It is unclear how stimulation of the lateral olfactory tract might alter AON interneuron activation and how this may differ from more specific activation of AON microcircuits after exposure to conspecific odour combinations. Vasopressin signalling in other brain regions may also impact AON function. Mice mothers given a central infusion of a V1aR antagonist show increased activation of the AON, as measured by fMRI blood oxygen level-dependent contrast imaging (aka BOLD), when exposed to a male intruder (Caffrey et al. 2010). As it is unlikely that infused vasopressin penetrated to the AON, these effects may have been mediated via a series of processing steps, likely involving vasopressin-sensitive areas more closely situated to the ventricles. However, since fMRI BOLD assesses blood flow, it is also possible that the V1aR antagonist caused changes in the cerebral vasculature, resulting in the observed behavioural effects and/or activation of the AON. Regardless of mechanism, vasopressin signalling within the AON seems to be involved in the processing of conspecific cues. Information from the AON can then be transmitted to a number of brain regions, including the nearby PIR.

### Piriform cortex

The piriform cortex (PIR) is a three-layered cortex that lies caudal to the AON. It is divided into anterior and posterior regions, with the anterior region receiving widespread input from the AON and the posterior region being involved in integrating information from the anterior PIR and other brain regions to construct a perception of odour quality (Bekkers and Suzuki 2013; Gottfried et al. 2006; Hagiwara et al. 2012; Pitkanen 2000). The PIR receives inputs from a number of brain regions, including the MOB, endopiriform nucleus (En) and nLOT (Behan and Haberly 1999; Bekkers and Suzuki 2013; Brunjes et al. 2005; Haberly and Price 1978; Neville and Haberly 2003; Wilson 2008). It sends outputs to the MOB, AON, OT, multiple amygdalar areas including the MeA and COA and other higher processing regions. Vasopressin is expressed across both anterior and posterior parts and in all three layers of the PIR of male and female rats, in both GABAergic interneurons and, most extensively, in glutamatergic neurons in layer II (Tsuji et al. 2017a). Unilateral stimulation of the rat lateral olfactory tract (biphasic pulse, 50 Hz, 10 min) induces an increase in the immediate early gene product Fos in vasopressin-producing pyramidal output neurons in layer II, suggesting that vasopressin outputs from this region are involved in the propagation of olfactory information.

V1aR and V1bR mRNA is expressed and V1aR binding has been demonstrated in the PIR of male and female rats (Corbani et al. 2017; Dumais and Veenema 2016; Szot et al. 1994). Interestingly, while the number of vasopressin cells is not sexually dimorphic in the rat PIR, males show greater V1aR binding than females (Dumais and Veenema 2016; Tsuji et al. 2017a). However, V1aR binding does not differ between female rats in estrus vs. non-estrus phases, leaving open the possibility that androgens may be involved in the aforementioned sex differences in this region (Dumais and Veenema 2016). Unlike in the AON, most vasopressin cells in the PIR do not co-express V1aR or V1bR in male and female rats (Tsuji et al. 2017a; Wacker et al. 2010, 2011). Vasopressin’s widespread distribution across the PIR may mean that it plays multiple regulatory roles. Future studies should examine differences between vasopressin interneurons and principal neurons to clarify the function or functions of vasopressin release from these cells. For example, it is unclear whether release from these different cell types serves the same or disparate functions within the different layers of the PIR.

The endopiriform nucleus (En), closely associated with the PIR, sends and receives information from the MeA and COA and represents an area of potential integration between the main and accessory olfactory processing pathways (Pitkanen 2000). It projects to the OT, PIR, AON and nLOT (Behan and Haberly 1999; Cádiz-Moretti et al. 2017). V1aR binding is prevalent in the dorsal En of prairie and montane voles (Wang et al. 1997) and coppery titi monkeys (Freeman et al. 2014) but has not been reported in the rat. Vasopressin binding has been described in the En of the golden hamster (Szot et al. 1990) but this may represent binding to oxytocin receptors (Dubois-Dauphin et al. 1992).

### Olfactory tubercle

The olfactory tubercle (OT), an amalgam of multiple functional regions, including the islands of Calleja (IC), is located medial to the PIR (Ikemoto 2010; Kruger et al. 1995; Wesson and Wilson 2011). It comprises regions connected to/from both the mesolimbic reward system and areas associated with multiple sensory modalities and recent evidence suggests it plays a role in coding the salience of odour stimuli (Ikemoto 2007; Scalia and Winans 1975; Scott et al. 1980; Wesson and Wilson 2011; Yamaguchi 2017). Neurons in the OT increase their firing rates and spike over a longer period of time when male mice are presented with an odour previously associated with a reward (Gadziola et al. 2015). Inhibition of neurons in the medial OT by the DREADD receptor, hM4Di, eliminates the preference of female mice for male versus female urine (DiBenedictis et al. 2015).

The OT, especially medial aspects, is sensitive to vasopressin. Vasopressin-ir fibres have been described in medial aspects of the OT in male rats and male and female mice (de Vries and Miller 1999; Otero-Garcia et al. 2014). V1aR binding and mRNA expression have been described in the rat medial OT (Ostrowski et al. 1994; Veinante and Freund-Mercier 1997). Though, Tribollet et al. (1988) only reported minimal vasopressin binding in this region, attributing it to vasopressin binding of oxytocin receptors. Szot et al. (1994) described V1aR mRNA within the IC in male and female Long-Evans rats but Ostrowski et al. (1994) did not detect hybridization within this region in their extensive analysis of male Sprague-Dawley rats. Hernando et al. (2001) reported V1bR-immunoreactivity in the OT, including rostral aspects of the IC, in male rats administered colchicine. The functional relevance of vasopressin signalling in the OT, whether by vasopressin or oxytocin receptor activation, requires additional study. There is evidence that vasopressin has at least indirect effects on OT function via V1aR activation, as it shows a reduction in activation in response to a noxious odour, butyric acid, when animals are pre-treated with a central infusion of a V1aR antagonist (Reed et al. 2013).

Vasopressin in the OT is likely supplied, at least in part, by projections from the medial extended amygdala (de Vries and Miller 1999, Otero-Garcia et al. 2014). The MeA and related BSt are important integrators in the social behaviour network, a collection of interconnected brain regions that modulate vertebrate social behaviour (Albers 2015). A connection between the MeA and the OT represents another link between this network and the mesolimbic reward system and therefore compromises a potential hub for sensory/behavioural integration in the larger social decision-making network (O'Connell and Hofmann 2011).

### Locus coeruleus

The locus coeruleus (LC), a nucleus in the pons of the hindbrain, sends noradrenergic inputs to the olfactory bulbs, AON, PIR and OT (Fallon and Moore 1978; Shipley et al. 1985). A number of neurons in the LC, including noradrenergic neurons, are immunopositive for vasopressin (Todoroki et al. 2010; Caffe et al. 1985). Vasopressin can modulate the activity of LC neurons, thus altering norepinephrine release (Gardner et al. 1984; Olpe and Baltzer 1981). Like vasopressin, norepinephrine is involved in olfactory learning in rats (Dluzen et al. 1998; Sullivan et al. 2000; Sullivan et al. 1994). So, it is possible that vasopressin within the LC elicits some of its effects on olfactory learning by modulating norepinephrine release. It is yet unclear whether vasopressin from the LC is released directly into the olfactory bulbs. The LC projects to multiple areas of the MOB and AOB, including the external plexiform layer and mitral cell layer, location of the bulbar vasopressin neurons and one of their putative targets respectively (McLean et al. 1989; Tobin et al. 2010). Bilateral infusions of vasopressin into the olfactory bulbs prior to a social recognition test increase the retention interval for social memory of a juvenile to 120 min in male rats via a V1aR signalling mechanism (Dluzen et al. 1998; Tobin et al. 2010). Depletion of norepinephrine by 6-OHDA blocks this facilitation (Dluzen et al. 1998). This, along with the observation that bulbar vasopressin administration increases norepinephrine release in multiparous ewes, suggests a synergistic relationship between norepinephrine and vasopressin in the olfactory bulbs (Levy et al. 1995).

### Amygdala/bed nucleus of the stria terminalis

The amygdala and related bed nucleus of the stria terminalis (BSt) are multi-faceted interconnected structures with a number of reciprocal connections with olfactory processing areas and other brain regions (Janak and Tye 2015; Pitkanen 2000; Swanson and Petrovich 1998). As hubs in multiple brain networks, these regions are responsible for a multitude of functions, the extensive treatment of which is beyond the scope of this paper. However, with respect to vasopressin modulation of olfactory processing, there are a number of important points to consider. First, there are vasopressin neurons in the medial extended amygdala, which includes the MeA and BSt. These neurons project to a number of brain regions including the lateral septum (LS) and the ventromedial IC of the OT (Otero-Garcia et al. 2014). Neurons of the MeA/BSt are often sexually dimorphic, with greater numbers in male animals and castration of males causes a pronounced decrease in vasopressin-ir in these regions and its targets in multiple vertebrate species (Aste et al. 1997; Miller et al. 1992; Van Leeuwen et al. 1985; Wang and De Vries 1993). Androgen receptor knockout mice do not show deficits in vasopressin mRNA expression in BSt or vasopressin-ir in the LS, strongly suggesting that local conversion of androgens to 17β-oestradiol by the enzyme aromatase is required for sex steroid-induced changes in these regions (Marie-Luce et al. 2013). This is consistent with previous work showing a reduction in vasopressin immunoreactivity in cells in BSt and fibres in LS in aromatase knockout rats (Plumari et al. 2002) and the reversal of castration-induced decreases in vasotocin-ir in the BSt and LS of male Japanese Quail with oestrogen administration (Viglietti-Panzica et al. 2001). Many regions of the BSt and subdivisions of amygdala are sensitive to vasopressin, including the COApm, COApl, MeA and central amygdala (Dumais and Veenema 2016; Ostrowski et al. 1994; Vaccari et al. 1998; Veinante and Freund-Mercier 1997), though continued comparative analyses are needed to determine the extent to which binding is species-specific and sexually dimorphic across taxonomic groups. Receptor binding in the medial extended amygdala and its targets can be sexually dimorphic; for example, there is greater binding in males in the medial BSt but interestingly not the LS of rats (see Dumais and Veenema 2016 for a more extensive cross-species review of sexual dimorphism in vasopressin signalling systems).

The amygdala represents a location for potential integration of signals between the main and accessory olfactory systems. The COAa (main olfactory system) and MeA (accessory olfactory system) are interconnected (Cádiz-Moretti et al. 2017; Pitkanen 2000) and inputs from the AOB and COA co-modulate neuronal firing at the level of the posterior ventral subdivision of the MeA (Keshavarzi et al. 2015). The MeA/BSt is a hub in the social decision-making network and represents a conduit by which olfactory information can influence context-dependent social behaviour (Bester-Meredith et al. 2015; O'Connell and Hofmann 2011). Vasopressin signalling at MeA/BSt targets, like the LS, is involved in the modulation of social behaviour across many species in both males and females, from social odour recognition to pair bonding to aggression (Bester-Meredith and Marler 2001; Bielsky et al. 2005; Goodson and Wang 2006; Liu et al. 2001; Veenema et al. 2012; Veenema et al. 2010). For example, vasopressin-ir neurons in the posterior medial BSt show a significant increase in activation, as assessed by the immediate early gene product Fos, after copulation in male mice (Ho et al. 2010). In a comparison of multiple species of male and female finches, Goodson and Wang (2006) found more vasotocin-ir cells in the medial BSt of socially affiliative vs. territorial species and that gregarious species show a greater activation of those cells after exposure to a same-sex conspecific. However, results proved more complex in one highly territorial and notoriously aggressive species, the violet-eared waxbill. While activation of vasotocin neurons in the BSt decreased when waxbills were exposed to same-sex conspecifics, activation actually increased when an animal was exposed to its mate. This suggests that a species’ natural history should be carefully considered when comparing vasopressin signalling across species. Such examples are not restricted to birds. Male Syrian hamsters show much longer recognition of conspecific flank gland odours than male rats show to conspecifics in social recognition or habituation/dishabituation tests (Ferguson et al. 2002; Song et al. 2016). Flank gland odour recognition is mediated by central oxytocin receptor activation, unlike social recognition in male rats, which involves V1aR activation in the LS and MOB (Bielsky et al. 2005; Landgraf et al. 1995; Song et al. 2016; Tobin et al. 2010). Species-level differences in vasopressin signalling between animals in the same genus are also well characterised (Bester-Meredith et al. 1999; Dewan et al. 2008; Insel et al. 1994) and vasopressin systems can also change in individuals across different life history stages, which is especially apparent in seasonally breeding animals (Goodson et al. 2012; Hermes et al. 1990).

Vasopressin-signalling systems can also change with social environment during development (Bester-Meredith et al. 1999; Grundwald et al. 2016; Yohn et al. 2017). For example, male and female California mice who were more often retrieved by their fathers as pups show both higher aggression and a greater number of vasopressin-ir cells within the BSt as adults (Yohn et al. 2017). Adult female rats whose mothers were exposed to stress during late pregnancy show reduced V1aR mRNA expression in the LS and BSt and have deficits in social odour memory after a 3-h retention interval in a social discrimination test (Grundwald et al. 2016). There are also changes in vasopressin signalling machinery observed during adult development, with fatherhood eliciting changes in multiple species (Bamshad et al. 1993; Lambert et al. 2011; Perea-Rodriguez et al. 2015). Experience and condition-induced changes in vasopressin signalling are often seen in sexually dimorphic regions like the BSt but changes in other regions have also been reported. More work is needed to determine whether there are seasonal and social condition-induced changes in vasopressin signalling in areas where vasopressin neurons have been more recently described, such as the olfactory bulbs, AON and PIR.

The LS, a primary target of MeA/BSt vasopressin neurons, is involved in olfactory-based short-term social recognition in rodents. Application of a V1aR antagonist into the LS but not the MeA, of male mice reduces habituation to an ovariectomized female in a habituation/dishabituation test (Bielsky et al. 2005). Male V1aR gene knockout mice show severe deficits in this test, which can be rescued with induced viral-mediated V1aR expression into the LS alone (Bielsky et al. 2004; Bielsky and Young 2004). However, Wersinger et al. (2007) demonstrated that male V1aR gene knockouts show normal social recognition with the same habituation/dishabituation test design, though these animals did show reduced olfactory investigation of novel social (urine) and non-social (almond scent) stimuli. These contradictory findings have been previously reported but their underpinnings remain unclear. Considering that prenatal stress can induce changes in oxytocin and vasopressin signalling systems and subsequent social behaviour in adult male and female rats (de Souza et al. 2013; Grundwald et al. 2016) and that vasopressin can bind and activate oxytocin receptors (Song and Albers 2017; Song et al. 2016), it is possible that varying developmental experiences led to different compensatory neural mechanisms in these animals, perhaps involving oxytocin receptor signalling. Similar vasopressin/oxytocin interactions were recently demonstrated in male oxytocin knockout mice, which show increased V1bR expression in the hippocampus and reduced vasopressin expression in the hypothalamus, as well as altered social behaviour (Lazzari et al. 2017). It is becoming clear that the social environment during development and adulthood must be considered when comparing the behavioural relevance of vasopressin signalling, even within the same model species.

## Vasopressin and visual/light processing

In mammals, light is transduced by two sets of photoreceptive cells in the retina, the rods and cones and the intrinsically photoreceptive retinal ganglion cells, or ipRGCs (Berson et al. 2002; Sterling and Demb 1990). This gives an animal two lines of information, one used for processing the identification, location, movement, etc. of stimuli within the visual field and another for processing information about time of day and day length to entrain circadian rhythms to properly match morphology, physiology and behaviour with predictable environment change. Visual information received by rods and cones is funnelled through a variety of bipolar cells modulated by horizontal and amacrine cells and sent towards the brain by multiple types of ganglion cell (Ghosh et al. 2004; Stephan and Zucker 1972; Sterling and Demb 1990). This information is then processed by and transmitted through the lateral geniculate nucleus (LGN) of the thalamus to a series of cortical processing areas, where perception of visual stimuli is refined (Priebe and McGee 2014; Sherman and Koch 1986). Light information received by ipRGCs is transmitted directly to the ventrolateral SCN to facilitate photoentrainment and to the olivary pretectal nucleus (OPN) to mediate the pupillary light reflex (Antle et al. 2009; Clarke and Ikeda 1985; Gall et al. 2017; Hattar et al. 2002; Morin 2013). The retina also projects to the superior colliculus (SC) to coordinate proper eye movements and gaze shifts (Klier et al. 2001; Lee et al. 1988). Vasopressin and its receptors are expressed at a number of locations in the visual and light information processing pathways and plays a critical function in the modulation of circadian rhythmicity.

### Retina

Vasopressin-ir cells were described in the ganglion cell layer of the rat retina over 20 years ago but received little attention until recently (Djeridane 1994). Tsuji et al. (2017b) characterized these neurons as a subset of glutamatergic retinal ganglion cells (VP-RGCs) in male and female rats. VP-RGCs show an upregulation of the immediate early gene product Fos in response to light, with some neurons activated and others inhibited by this stimulus. Some, but not all, VP-RGCs express melanopsin, so while a subset of these cells are inherently photosensitive, others likely receive input from nearby ipRGCs. VP-RGCs project to the intergeniculate leaflet (IGL), OPN and ventrolateral SCN, supplying important information about light exposure to these regions.

### Suprachiasmatic nucleus

The hypothalamic suprachiasmatic nucleus (SCN) is the location of the mammalian master clock, which coordinates peripheral clocks and facilitates circadian physiology, morphology and behaviour (Antle and Silver 2005; Stephan and Zucker 1972). The SCN is typically subdivided into a dorsomedial shell and ventrolateral core, with the ventrolateral region receiving most but not all, of the retinal input (Antle et al. 2009; Morin 2007). This organization, however, is species-specific and other methods of defining SCN subdivisions have been proposed (Campos et al. 2014; Morin 2007). Neurons in the ventrolateral core receive and transduce light information into the rhythmic expression of clock genes to facilitate photoentrainment (Antle and Silver 2005). This information is integrated and passed along to the inherently rhythmic dorsomedial shell, which is populated by vasopressin neurons (Antle and Silver 2005; Evans et al. 2015; Morin 2013).

Vasopressin-ir cells in the SCN help orchestrate circadian rhythmicity (Kalsbeek et al. 2010; Kalsbeek et al. 2006; Sofroniew and Weindl 1980). As vasopressin neuron number, development of vasopressin expression and V1aR binding is not sexually dimorphic, the SCN does not appear to be sensitive to gonadal steroids like the extended medial amygdala (De Vries and Panzica 2006; Dumais and Veenema 2016; Smith et al. 2017; Swaab et al. 1985; Szot and Dorsa 1993). Vasopressin neurons in the SCN project to multiple neural areas in the rat, including the dorsomedial nucleus of the hypothalamus (DMH), LS, organum vasculosum laminae terminalis (OVLT) and PVN (Buijs 1978; Hoorneman and Buijs 1982; Sofroniew and Weindl 1978). Campos et al. (2014) described similar projections from the SCN to the DMH and PVN in the tufted capuchin monkey and also additional connections to other hypothalamic regions. Within the SCN, vasopressin mRNA expression and neuronal firing cycle, with peaks during the day (Cagampang et al. 1994; Kalsbeek et al. 2010; Uhl and Reppert 1986; Young 3rd et al. 1993). Vasopressin levels show similar cycles in the cerebrospinal fluid as well (Reppert et al. 1981). Vasopressin secretion within the SCN also cycles, with increases during the day and it is yet unclear whether these peaks in secretion represent somato-dendritic release from the SCN itself and/or axonal input from VP-RGCs (Kalsbeek et al. 1995; Tsuji et al. 2017b). The SCN contains V1 receptors, upon which vasopressin can act to modulate neuronal firing (Mihai et al. 1994; Phillips et al. 1988; Swaab et al. 1975; Szot et al. 1994; Vaccari et al. 1998). V1aR mRNA expression and vasopressin induced neuronal excitation within the SCN cycles, with peaks in the night, differing from the daytime peaks as seen for vasopressin mRNA (Liou and Albers 1989; Young 3rd et al. 1993). Vasopressin also regulates targets outside of the SCN to help orchestrate circadian physiology and behaviour (Gizowski et al. 2016; Mihai et al. 1994; Miller et al. 2006). ICV injections of vasopressin rescue a normal luteinizing hormone (LH) surge in female mice with mutant CLOCK genes (i.e., Clock/Clock mutants) (Miller et al. 2004, 2006) and vasopressin secretion from the SCN is required for properly timed glucocorticoid release in male rats (Kalsbeek et al. 1996). However, microinjection of vasopressin directly into the SCN does not reset the circadian clock in male hamsters or rats (Albers et al. 1984, Arnauld et al. 1989). Vasopressin output from the SCN also regulates non-endocrine targets. For example, vasopressin release from SCN neurons activates cells in the OVLT to induce pre-sleep thirst in mice to prevent night-time dehydration (Gizowski et al. 2016, 2018).

Studies examining V1R signalling have further highlighted vasopressin’s critical role in maintaining proper circadian rhythmicity. Intracerebroventricular injections of a V1aR antagonist reduce both light-induced and retino-hypothalamic tract stimulation-induced firing of SCN neurons of male and female rats (Tsuji et al. 2017b). While male and female V1aR gene knockout mice maintain normal locomotor rhythms on a 12:12 light dark cycle, they increase their activity when placed in constant darkness, with some animals eventually becoming arrhythmic (Li et al. 2009). Mice that lack both V1R subtypes entrain to new light dark cycles in less than half the time of wild-type animals, suggesting that vasopressin normally acts to slow photoentrainment (Yamaguchi et al. 2013).

### Other visual/light processing areas

The actions of vasopressin in the SCN have received much more attention than those in other visual/light processing regions. The SC, LGN, IGL and OPN all receive vasopressin input and/or are sensitive to vasopressin in at least some species. The SC receives retinal input from a variety of retinal ganglion subtypes, integrating this information with that from other brain areas and sensory systems to facilitate appropriate eye and gaze movements (Gandhi and Katnani 2011; Lee et al. 1988). Vasopressin binding has been demonstrated in the SC of a number of species, including male and female rats, prairie voles and montane voles (Insel et al. 1994; Phillips et al. 1988; Smith et al. 2017). Compared to other vasopressin-sensitive brain regions, there is a relatively high level of inter-individual variation in vasopressin binding in the superior colliculi of lab-reared prairie voles but the function of this difference is yet unclear (Hammock et al. 2005). Unlike the SCN, the SC does not receive projections from VP-RGCs, rather from SCN neurons (Buijs 1978; Tsuji et al. 2017b).

The dorsal LGN receives and processes input from retinal ganglion cells and sends visual information on for further processing by visual cortical areas (Monavarfeshani et al. 2017). Vasopressin binding has been demonstrated in the LGN of a few species, including male and female coppery titi monkeys and singing mice (Campbell et al. 2009; Freeman et al. 2014). Receptor binding intensity was shown to vary across two different species of singing mice within the Genus Scotinomys but the functional relevance of this difference and vasopressin binding within the dorsal LGN more generally, has yet to be examined in great detail (Campbell et al. 2009).

The OPN receives input from VP-RGCs (Tsuji et al. 2017a, b). This nucleus helps mediate the pupillary light reflex, as lesions to the OPN prevent this response (Clarke and Ikeda 1985; Gall et al. 2017). Since ipRGCs mediate at least some of the input underlying the pupillary light reflex, it would be helpful to know whether and how VP-RGCs contribute to this response at the level of the OPN (Barrionuevo and Cao 2016; Tsuji et al. 2017b). The IGL also receives input from VP-RGCs. This region shares connections with the OPN, SCN, SC and the LC (another source of neural vasopressin) and sends projections to the DMH (also a SCN target) and PVN (Monavarfeshani et al. 2017; Moore et al. 2000). IGL lesions in female diurnal grass rats lead to a reduction in neuronal activation in the OPN when animals are given a light pulse during the dark phase of a 12:12 cycle (Gall et al. 2014). The presence of ipRGC input and other physiological evidence suggests that the IGL is involved in photoentrainment, though it is clear that the SCN is the primary mediator of circadian rhythmicity (Edelstein and Amir 1999; Gall et al. 2014; Monavarfeshani et al. 2017).

## Integration of vasopressin signalling across neural pools

Vasopressin is produced and signals, in multiple sensory and higher brain processing areas, many of which are interconnected. Here, we reviewed vasopressin’s role in the processing of olfactory, pheromonal and visual/light information. Other authors have outlined how this neuropeptide modulates the processing of auditory and other sensory cues and yet others have described vasopressin’s well-known actions mediating affiliative and agonistic social behaviours in a multitude of vertebrates (Albers 2015, Bester-Meredith et al. 2015, Goodson and Bass 2001, Rose and Moore 2002, Wilczynski et al. 2017). Despite the connections between many vasopressin-producing and vasopressin-sensitive areas, its actions can be discrete and its functions region-specific. For example, vasopressin released from neurons in the olfactory bulbs has primarily local effects (though there is some evidence for scant projections to the AON) (Tobin et al. 2010). Expression and binding of vasopressin is sexually dimorphic and dependent on gonadal steroids in some brain regions (e.g., vasopressin-ir cell number in rat BSt; binding in the ventrolateral hypothalamus in hamsters) but not in others (e.g., vasopressin-ir cell number in olfactory bulbs, AON and PIR), suggesting brain region-specific modulation of vasopressin signalling (Delville and Ferris 1995; Dumais and Veenema 2016; Miller et al. 1992; Tobin et al. 2010; Tsuji et al. 2017a; Van Leeuwen et al. 1985; Wacker et al. 2010). Still, vasopressin signalling across multiple neural networks is critical for the integration of social and environmental stimuli and context-appropriate social behaviour. One excellent example of multiple vasopressin systems coordinating to modulate behaviour involves short-term social recognition in rodents. Vasopressin produced by neurons in the MOB acts locally and vasopressin produced by neurons in the MeA/BSt acts within the LS to mediate such recognition (Bielsky et al. 2005; Tobin et al. 2010). These two vasopressin signalling pools are thought to be segregated but both function to mediate appropriate behavioural responses in recognition tests.

The extent to which vasopressin from different source pools might interact *within* brain regions, especially extrahypothalamic areas, to mediate changes in social discrimination and other behaviours has not yet been fully explored. For example, it is possible that separate populations of vasopressin-producing neurons in the MOB, AON, PIR and LC co-modulate vasopressin signalling in the MOB. Such signalling could be elicited via different mechanisms, as is the case for axonal vs. somato-dendritic vasopressin release from magnocellular neurons in the SON (Ludwig and Leng 2006; Ludwig and Stern 2015). This might allow for a fine tuning of responses, involving multiple signalling centres. Neuronal tract tracing, immunohistochemical labelling and electron microscopy could be combined to establish whether axonal projections to the olfactory bulbs from these areas contain vasopressin (Landry et al. 2003). For example, it would be helpful to know how frequently vasopressin is co-localized with norepinephrine in axons of LC neurons in the olfactory bulb, or whether the putative connection from the MeA to the olfactory bulbs contains vasopressin. The potential for such integrative signalling is not restricted to the olfactory bulbs. For instance, it is yet unclear the extent to which SCN and retinal vasopressin might co-modulate circadian function within the SCN itself.

Vasopressin released from multiple neural sources acts at a variety of targets (both local and distant) by activating V1aR, V1bR and oxytocin receptors to mediate changes in olfactory and light/visual processing and social behaviour. Vasopressin signalling systems sometimes show differences associated with the natural history of the species examined, developmental stress, social condition and the ebb and flow of gonadal steroids across annually repeating life history stages. Some vasopressin signalling pools are sexually dimorphic, while others are not. All of these factors must be considered to achieve a more complete understanding of vasopressin’s role in sensory processing and behavioural modulation.
